# Toxicity Research of PM_2.5_ Compositions in Vitro

**DOI:** 10.3390/ijerph14030232

**Published:** 2017-02-26

**Authors:** Yi-Yang Jia, Qi Wang, Te Liu

**Affiliations:** 1 Scientific Research Center, China-Japan Union Hospital of Jilin University, Changchun 130033, China; 2 Department of Occupational and Environmental Health, School of Public Health, Jilin University, Changchun 130021, China; jiayy14@mails.jlu.edu.cn (Y.-Y.J.); wangqi15@mails.jlu.edu.cn (Q.W.)

**Keywords:** cell, particular matter, components, toxicology, health

## Abstract

According to the published literature, we surmise that particulate matter (PM) concentration, individually, may be less important than components in explaining health effects. PM_2.5_ (aerodynamic diameter < 2.5 μm) had similar cytotoxicity (e.g., cell viability reduction, oxidative damage, inflammatory effects and genetic toxicity) on different types of cells. The studies of cells are readily available for detailed mechanistic investigations, which is more appropriate for learning and comparing the mechanism caused by single or mixed ingredients coating a carbon core. No review exists that holistically examines the evidence from all components-based in vitro studies. We reviewed published studies that focus on the cytotoxicity of normal PM_2.5_. Those studies suggested that the toxicity of mixed compositions differs greatly from the single ingredients in mixed components and the target cells. The cytotoxic responses caused by PM_2.5_ components have not shown a consistent association with clear, specific health effects. The results may be beneficial for providing new targets for drugs for the treatment of PM_2.5_-related diseases.

## 1. Introduction

With the rapid growth of economy and industry, the emissions of air pollutant sources have made air quality a crucial health problem. According to the 2010 Global Burden of Disease Study, ambient air pollution caused more than three million deaths per year, which is the ninth leading factor contributing to the worldwide burden of disease [1]. Particulate matter (PM) is an important part of air pollution, and is an air-suspended mixture of solid and liquid particles. Currently, major sources of ambient PM include vehicular and industrial emissions, power plants, refuse incineration, and geological material. PM can be classified as PM_10_ (aerodynamic diameter < 10 μm), PM_2.5_ (aerodynamic diameter < 2.5 μm), and PM_1_ (aerodynamic diameter < 1 μm). Studies have shown that the smaller the diameter of particles, the greater the harm to human health. Ultrafine particle matter (UPM, PM < 0.1 μm) can even enter cells and cause direct damage to macromolecules [2]. PM_2.5_, which is regulated under the National Ambient Air Quality Standards (NAAQS) and is considered as an indicator of air pollution [3], is supposed to be more harmful due to its smaller dimension and its ability to distribute into the lung’s alveolar districts. The physicochemical characteristics of PM_2.5_ samples revealed their high heterogeneities and complexities related to the multiple natural and anthropogenic emission sources [4].

At present, a large number of scholars—through epidemiological and animal models—have confirmed the toxic effects of PM_2.5_ on human and animal health. However, epidemiological studies show statistical associations between health outcomes and exposure, but they cannot establish a definite cause–effect relationship. The utility of toxicological studies is to establish this relationship. To further research mechanisms of toxicity, some scholars have conducted PM_2.5_ experimental studies from the cellular and molecular levels. In vitro human cell models are more or less representative of various cell types, in relation either to the embryological origin or to the tissue or organ from which they have been derived [5]. The cells of study are readily available for detailed mechanistic investigations, and species-to-species extrapolations can be avoided since human cells are most often used.

The published studies show that the effect of PM on human health is determined by the size of and the contaminants adsorbed on the particles, which has a causal relationship with multiple health endpoints [6,7]. In addition, some studies show that the PM composition may vary considerably depending on sampling season, region, and sources [8]. PM_2.5_ samples in urban areas were chemically characterized for inorganic ions, total carbon (including OC—organic carbon and EC—elemental carbon), elements (mineral dust and trace elements from anthropogenic sources), polycyclic aromatic hydrocarbons (PAHs), and biological products (endotoxin). Numerous studies and reviews focus on the correlations between PM_2.5_ components and health effects and proved it can be high.

Current knowledge does not allow precise quantification of the health effects of individual PM components or of PM emissions from different sources [9]. Generally, cells or organisms are exposed to extracts of the particles rather than the native particles in order to study the effects of inorganic elements (i.e., Fe, Al, Ca, Na, K, Mg, Pb, etc.), inorganic ions (i.e., Cl^−^, F^−^, SO_4_^2−^, NO_3_^−^, etc.), and organic substances (i.e., volatile organic compounds (VOCs), PAH, etc.). Water-soluble inorganic ions and endotoxins were extracted in pure water, and inorganic ions were analyzed by ionic chromatography. Organic substances were extracted with organic solvents (dichloromethane, acetonitrile, etc.). Organic and water-soluble extracts of PM_2.5_ were analyzed to quantify PAHs by GC-MS (gas chromatography-mass spectrometry) and HPLC-FD (High performance liquid chromatography fluorescence detection) technique and metals by the ICP-MS (inductively coupled plasma mass spectrometer) technique. In addition, TiO_2_, thermally desorbed PM_2.5_ (dPM), and CB (carbon black) concentrations were in most cases regarded as negative controls to identify the effects of components adsorbed on PM_2.5_ or its carbon core. Health effects caused by metal and PAH (and other organic compound)-enriched particles are likely to be primarily responsible for their high redox capacity [10,11]. PAHs are metabolized by the Cytochrome P450 (CYP) superfamily member CYP1A1 [10,11] and require metabolic activation by biologically reactive intermediates to elicit their adverse health effects. The PAHs are absorbed and distributed into lung cells and/or tissues, where they can be bio-transformed. Due to their high heterogeneities and complexities, the mechanisms of chemical constituents that cause harmful health effects are largely unclear. Much more knowledge is needed if we want to identify and characterize the specific health effects of each component.

Studies published on the health impacts of PM_2.5_ constituents have substantially enhanced our knowledge and have clearly filled some major knowledge gaps. However, there are no reviews that holistically examine the evidence from all component-based studies in vitro. Here, we summarize the in vitro toxicological research on the health effects of the main PM_2.5_ components in recent years, finding the corresponding relationship between different components and specific cell damage, and elucidate the possible biological mechanisms involved. The results of this review provide a basis for guiding future research on acute and chronic adverse health impacts caused by PM_2.5_.

## 2. Evaluation Approach and Summary of Methods

We conducted a comprehensive evaluation of in vitro toxicological studies that examined groups of PM_2.5_ components. In the text below, studies have been grouped by cell types for clarity and ease of reading. We used PubMed, entering search terms of “cell”, “PM_2.5_”, “particulate matter”, “air pollution”, “ultrafine particles”, “components”, “toxicology”, and “health”, in various combinations. Of the total papers identified, we applied a specific inclusion criteria, as follows:

(a) Focused on normal ambient PM_2.5_, not surrogate particles such as residual oil fly ash (ROFA);
(b) Toxicology studies had to include cells (therefore, acellular assays were excluded);
(c) Included at least one PM component, both single compound and co-pollutants were considered; and
(d) Formal statistical analysis investigating the relationship between groups of PM components and health effects.

In this review, we focus only on normal PM_2.5_. In addition, including all three disciplines at the same time enabled greater integration of findings from a number of locations, which would be severely limited if only one discipline were considered.

## 3. Findings

### 3.1. Toxicity Studies on Lung Adenocarcinoma Cells (A549)

A549 cells are derived from human alveolar cell carcinoma of lung cancer cells with both characteristics of malignant tumor cells and alveolar type II cells. Therefore, the A549 cells are widely used in the research on lung cancer’s development, diagnosis, and treatment, or normal human alveolar type II cell’s differentiation, structure, and functional research. The study conducted by Pavagadhi et al. examined the biological effects of the PM_2.5_ samples of Singapore on human lung epithelial cell line A549. The PM_2.5_ samples were analyzed for 16 priority PAHs and 10 transition metals. Its biological effects include a decrease in cell viability and an increase in cell death [12]. Billet et al. found that relatively low and the PM extract, suggesting that the PM components may doses of PAH-coated onto PM_2.5_ were able to significantly induce both the gene expression and the catalytic activity of CYP1A1 in A549 cells [10,11]. Alessandria et al. collected PM_2.5_ in Torino and evaluated the biological effects of aqueous and organic solvent PM extracts on human epithelial lung A549. They reported that PM_2.5_ extracts inhibited cell proliferation and induced lactate dehydrogenase (LDH) release in a dose-dependent manner. No significant correlations were found between the biological effects be as an entire mixture in inducing cytotoxic response [13]. However, Gualtieri et al. exposed the A549 cell line to PAHs and metals of Milan winter PM_2.5_. They observed cell membrane lysis and mitochondrial ultrastructural disruptions, and cell mortality and ultrastructural lesions were observed concomitantly. A significant intracellular production of reactive oxygen species (ROS) was also observed, suggesting that the cytotoxicity was probably associated with the oxidative potential of particle-adsorbed metals and PAHs [1]. Wang et al. also examined parent PAHs, nitrated PAHs (NPAHs) and oxygenated PAHs (OPAHs), and they reported that they all showed serious DNA damage on human A549 lung carcinoma cells with the daily PM extracts in the Comet assay. NPAH and OPAH concentrations were 8% of the parent PAH concentrations, while the direct-acting mutagenicity (due to the NPAH and OPAH) was 200% higher than the indirect-acting mutagenicity (due to the PAH). This result suggests that NPAH and OPAH make up a significant portion of the overall mutagenicity of PM_2.5_ [14].

Bonetta et al. found that the chemical analysis of the three different sites (urban, industrial, or highway sites) showed a variability of PAH composition in PM organic extracts and 14 metals (including Fe, Cu, Zn, Sb, and Ba the most abundant) in all of the PM water extracts. Regarding the biological effect, all of the PM_2.5_ organic extracts caused a significant increase of the A549 DNA damage in a dose-dependent manner. The genotoxic effect was related to the PM_2.5_ PAH content, and the highest effect was observed for the highway site sample (high ΣPAHs concentration). The DNA oxidative damages were observed for the PM_2.5_ water extracts of the samples collected in industrial and highway sites (high Σ metals concentration). The extent of the oxidative damage may be related to the concentration of the metals present [1].

Quinones are an important part of the organic extracts from fine particulate matter. Shang et al. examined the genotoxic and inflammatory impacts of organic extracts from traffic-related PM (oTRP) in human lung epithelial A549 cells, and revealed the contributions from quinines. Their results suggested that oTRP may mediate genotoxic and inflammatory effects through the oxidative stress pathway. Furthermore, the effects of two typical airborne quinones—9,10-anthraquinone (AQ) and 1,4-naphthroquinone (NQ)—were compared. NQ, but not AQ, induced significant DNA damage in A549 cells. NQ up-regulated interleukin 8 (IL-8), TNF-α, and Mcp-1 genes, while AQ induced the expression of the RANTES (regulated upon activation normal T-cell expressed and secreted) gene. These results suggest that the NQ and AQ may participate in the pro-inflammatory responses by releasing different types of cytokines/chemokines [15]. In order to further assess the cytotoxicity of each composition coated on fine particulate matter, Bourgeois et al. extracted metals, endotoxins, and PAH and introduced them to A549 human epithelial lung carcinoma cells. They found that aqueous PM_2.5_ extracts decreased cell viability in a dose-dependent manner, while endotoxins alone showed no cytotoxicity. On the contrary, the concentration of reactive oxygen species (ROS) and released LDH activity increased following aqueous PM_2.5_ extracts’ exposure to A549 cells. Regarding inflammatory response, all PM_2.5_ extracts up-regulated cytokines involved in the down-stream activation of the caspase cascade and kinase pathways, and the up-regulation of metal-redox-sensitive transcription factors NF-κβ and AP-1 is consistent with a cell death mechanism initiated by Fenton-active transition metal redox catalysis [16].

Perrone et al. investigated the metal-related composition of PM and their influence on some biological properties that appeared on the human lung carcinoma epithelial cell line A549. The highest correlation coefficients between cell viability reduction and single chemical components showed on As and SO_4_^2−^. The PM_2.5_ fraction—which was enriched in Ca^2+^ and Mg^2+^ and Al, Fe, Zn, Ba, and Mn—produced significant cell viability reduction and DNA damage [15]. Overall, through the studies on the composition of PM_2.5_ associated with A549 cell toxicity, it was shown that PM_2.5_ water-soluble extract, transition metals, and organic ingredients can all cause cytotoxicity, including decreasing cell activity and enhancing the inflammatory reaction and cytokine gene expression. In addition, damage to the cell membrane and cell ultrastructure of mitochondria is also one of the most important aspects of the cytotoxicity. We may be safe to conclude that organic ingredients—mainly PAHs—play a prominent role in genetic toxicity, and metal components offer a larger contribution to the oxidative damage. The combined effect of mixing extracts also cannot be ignored.

### 3.2. Toxicity Studies on Macrophages 

Alveolar macrophages play a major role in immune response to inhaled particles and are therefore a primary target for PM_2.5_ effects. They play an important role in swallowing particles and releasing cytokines, involved in almost every link of the immune inflammatory response. Daher et al. found that PM (2.5–0.25 μm), vehicular abrasion element Cu, and soil-derived component Co were highly associated with ROS activity by rat alveolar macrophage cells. In PM_0.25_, V and Ni originating from fuel oil combustion strongly correlated with ROS formation. Findings suggest a dominant role of transition metals in generating ROS compared to organic carbon [15].

Cavanagh et al. collected the PM_2.5_ from Auckland and Christchurch, New Zealand, and examined the response associated with their composition in RAW264.7 macrophages. PM collected from Christchurch had higher concentrations of PAH and water-soluble metal. The organic fraction was analyzed to examine PAH content, CYP1A1 induction, direct mutagenicity, TNF-α release, and cytotoxicity. The organic extracts of Christchurch PM_2.5_ showed higher mutagenicity and CYP1A1 induction compared with PM from Auckland. They also examined the metal content, cytotoxicity, and TNF-α release of the water-soluble fraction. In contrast, water-soluble extracts of Auckland PM (having lower metal content) were more cytotoxic and resulted in greater TNF-α release than those from Christchurch PM. The organic fraction of PM from both cities showed no cytokine release, and the organic extract from Auckland samples showed no cytotoxicity. Biological responses typically occurred at lower doses of the organic extract, indicating that water-soluble components may be less important in eliciting effects than organic components [15].

Jalava et al. focused on the effects of insoluble fractions of urban air fine particulate samples in addition to the water and organic-solvent-soluble particulate fractions. They investigated the inflammatory and cytotoxic activities of the water-soluble and insoluble, as well as organic-solvent-soluble. The experimental results show that both the water and organic-solvent-soluble particulate fractions increased TNF-α production release and apoptosis; however, insoluble components of the complex urban air particulate mixture induced the highest inflammatory and cytotoxic activities in macrophage cell lines. To a certain extent, they may serve as carriers for active water- and lipid-soluble components [15]. Pozzi et al. sought to study the biological effects of the organic compounds and endotoxin adsorbed on urban particles, as well as the particles’ core and transition metals by comparing the production of pro-inflammatory mediators in the monocytic–macrophagic RAW 264.7 cell line. The results indicated that transition metals play an important role in releasing both AA (arachidonic acid) and TNF-α. The organic compounds may induce cytokines and inflammatory mediator production [15]. Benzo[*a*]pyrene (B[a]P), Benzo[*b*]fluoranthene (B[b]F), and Pyrene (PYR) are quantitatively the most important ingredients of PAHs—the major organic compounds adsorbed onto these particles. Goulaouic et al. evaluated their impact on the cytokine production of THP-1 macrophage-like cells alone and/or adsorbed onto CB particles differing in size. The result can be concluded as PAH induced significant secretion of IL-1, IL-8, and IL-12 after 24 or 48 h of treatment; fine CB particles (260 nm diameter) induced the secretion of each cytokine, and the PAH coating the CB did not modify the effect of the CB alone. In contrast, ultrafine CB (14 nm diameter: ufCB) caused a decrease in cytokine secretion, and the effect was modified by the PAH coating [17]. Saint-Georges et al. treated alveolar macrophages (AMs) with VOC and/or PAH coated onto PM and the results showed an increase of the gene expressions of cytochrome P450 (cyp) 1a1 (cyp1a1), cyp2e1, cyp2f1, nadphquinone oxydo-reductase-1 (nqo1), microsomal epoxyde hydrolase (meh), mu3 (gst-μ3), and glutathione S-transferase-pi1 (gst-π1). In addition, these results suggested the carbonaceous core of PM can be the physical carrier of attached VOC and/or PAH to penetrate into the cells and remain, thus enabling them to exert a persistent induction. Hence, they concluded that the metabolic activation of the very low doses of VOC and/or PAH coated onto PM_2.5_ is one of the underlying mechanisms of action closely involved in cytotoxicity in isolated human alveolar macrophages in culture [18].

Steenhof et al. found the toxicity of ambient PM collected at different sites in the Netherlands in relation to PM composition and its oxidative potential in RAW264.7 cells. Fine and ultrafine particle samples were characterized by a high concentration of elemental carbon, organic carbon, and a high content of transition metals. The elemental carbon and organic carbon induced the highest pro-inflammatory activity, and the transition metals showed the largest cell viability decrease in MTT-reduction activity [19]. Shang et al. treated murine alveolar macrophages (MH-S) with PM_2.5_ samples collected before and during the control measures. Dose-dependent effects on cell viability, cytokine/chemokine release, and mRNA expressions were observed. As the result of Pearson correlation coefficients, significant associations were found for elemental zinc and viability in PM_2.5_, endotoxin content in PM_2.5_, and all of the eight detected cytokines/chemokines. Elemental and organic carbon correlated with four; arsenic and chromium correlated with six and three, respectively; iron and barium showed associations with two; and nickel, magnesium, potassium, and calcium showed associations with one. In the end, another significant association was found for viability and elemental zinc in PM_2.5_. In summary, PM_2.5_ toxicity in Beijing was substantially dependent on its chemical components [17]. Huang et al. determined the gene expression profile in human AMs exposed to particles, and the results showed that PM_2.5_ induced a gene expression profile prevalent with genes related to metal binding and oxidative stress in human AMs which was independent of oxidative stress. Metals coated on PM may play an important role in particle-induced gene changes [20].

Shafer et al. conducted a sensitive macrophage-based in vitro ROS assay induced by water-soluble metals in aerosol toxicity. They reported that transition metals—particularly iron—are major factors mediating ROS activity of water extracts of the Lahore PM. A small subset of metals (Mn, Co, Fe, Ni) were regarded as the potential ROS-active species, and several water-soluble “trace” metals (Zn, Pb, Cd) exhibited relatively poor correlations with ROS present at very high concentrations in the PM extracts [21]. Jalava et al. exposed mice for 24 h to PM (2.5–0.2 μm) samples from six European cities. The concentrations of pro-inflammatory cytokines (IL-6, TNF-α), chemokine (MIP-2), and nitric oxide were measured from the cell culture medium, and the cytotoxicity was assayed. Some transition metals (Ni, V, Fe, Cu, Cr) and insoluble soil constituents (Ca, Al, Fe, Si) correlated positively with the response parameters. In contrast, the tracers of incomplete biomass (monosaccharide anhydrides), coal (As) combustion, and PAHs had negative correlations with the inflammatory activity [22]. Doherty et al. treated alveolar macrophages with Fe alone and in conjunction with V, Mn, and/or Al at ratios of V:Fe, Al:Fe, or Mn:Fe encountered in PM_2.5_ samples from New York City, Los Angeles, and Seattle. To test this, iron-response protein (IRP) binding activity that would lead to altered antibacterial function was assessed. The results indicated that V and Al each significantly altered IRP activity, though effects were not consistently ratio (i.e., dose-) dependent; Mn had little impact on activity. We conclude that the reductions in Fe status detected here via the IRP assay arose in part from effects on transferrin-mediated Fe^3+^ delivery to the AM [23]. Prophete et al. determined whether any changes in AM IRP activity induced by PM_2.5_ constituents V, Mn, or Al were independent from effects of the metals on cell NO formation, then assessed for changes in IRP activity and iNOS expression. Besides, extracellular regulated protein kinases (ERK) 1 and 2 levels were also measured. The results indicate that V and Al—and to a lesser extent Mn—altered IRP activity, though the effects were not consistently concentration-dependent. Furthermore, while V and Mn treatments did not induce iNOS expression, Al did. These results confirmed the hypothesis that certain metals associated with PM_2.5_ might alter the pulmonary immunocompetence of exposed hosts by affecting the Fe status of AM—a major class of deep lung defense cells [24]. Based on the above studies, we can know that PM_2.5_ lipid-soluble components, water-soluble constituents, insoluble components, and carbon core can all cause damage to macrophages. Water-soluble organic ingredients (PAH, VOC, etc.) have a certain correlation with cell toxicity, gene expression, and mutation, the release of inflammatory factors and cytokines, etc. Metal fractions of water-soluble components can cause oxidative stress, the transition metals especially show quite prominent relations with inflammation, and metal components can affect the antibacterial ability of macrophages. The insoluble components and endotoxin are closely related with cytokine and the release of inflammatory factors. The interaction impacts between each ingredient may enhance or change the cell damage.

### 3.3. Toxicity Studies on Airway Epithelial Cells

In the respiratory system, the bronchial epithelium can protect the lungs by keeping it stable and playing the role of a mechanical barrier. However, in this process, the secretion of cytokines and other external stimuli can cause acute and chronic responses. The functional unit of human bronchial epithelial cells plays an important role, because the biological effects of bronchial epithelial cells in stress responses have important significance. Dieme et al. collected three air pollution PM_2.5_ samples in two urban sites and in a rural site, and PM_2.5_ samples source from urban sites caused greater biological responses than the rural one in BEAS-2B cells, in agreement with the physicochemical characterization. PM_2.5_ caused a time- and/or dose-dependent increase in both the gene expression and/or protein secretion of inflammatory mediators (i.e., IL-1, TNF-α, IL-8, and/or IL-6) in BEAS-2B cells. Variable concentrations of organic compounds (i.e., PAHs) and transition metals (i.e., Al, Fe, Mn, Pb, Zn) found in the three PM_2.5_ samples might be involved in a time- and/or dose-dependent toxicity relying on inflammatory processes [25]. 

Rumelhard et al. investigated the effect of Paris PM_2.5_ organic and aqueous fractions in amphiregulin expression and secretion on the bronchial epithelial cell line 16HBE and normal human nasal epithelial (NHNE) cells, and reported that both PM_2.5_ organic and hydrosoluble components are involved in the expression and secretion of AR, which is involved in repair responses [26]. 

Billet et al. reported that the VOC and/or PAH coated onto PM induced significant increases in mRNA expressions of cytochrome cyp2e1, cyp2f1, nadphquinone oxydo-reductase-1, P450 (CYP) 1a1, and glutathione s-transferase-pi1, versus controls. Based on this result, they concluded that the metabolic activation of VOC and/or PAH coated on the inorganic condensation nuclei is one of the underlying mechanisms involved in the cytotoxicity in human lung epithelial cells [25]. Baulig et al. investigated the contribution of each PM component to PM-induced biological effects by treating human bronchial epithelial cells with four samples of Paris PM_2.5_ having different poly-aromatic hydrocarbons and metals content, and their respective aqueous and organic extracts used alone or in combination. The PM-aqueous extracts contained soluble metals involved in hydroxyl radical production in abiotic conditions. However, they contributed slightly to the intracellular reactive oxygen species production release in comparison with organic extracts. Organic compounds transactivated the xenobiotic responsive element (XRE) and antioxidant responsive element (ARE), leading to increased cytochrome P450 1A1 expression and NADPH-quinone oxydoreductase-1 expression, respectively, but to different extents according to PM samples—underlying the differences in their bioavailability [27]. In order to further study the toxicity of organic compounds, Omura et al. further fractionated the dichloromethane-soluble fraction (DMSF) of diesel exhaust particles into a *n*-hexane soluble fraction (n-HSF) containing aliphatic and PAH and an *n*-hexane insoluble fraction (n-HISF) containing oxygenated compounds and strong oxidative properties. Rat alveolar epithelial (SV40T2) cells were exposed to DMSF, n-HSF, and n-HISF, and it was found that DMSF predominantly up-regulated genes associated with drug metabolism (Cyp1a1, Gsta3), oxidative stress response (HO-1, Srxn1), and cell cycle/apoptosis. Genes up-regulated by n-HSF were mainly associated with drug metabolism (Cyp1a1, Gsta3). The genes up-regulated by n-HISF included antioxidant enzymes (HO-1, Srxn1) and gene response to cell damage, such as gene-related regulation or apoptosis and genes in coagulation pathways. Their present results suggested that n-HSF and n-HISF regulated characteristic genes which responded to the chemical properties of each fraction [28]. 

Ferecatu et al. investigated the components of Parisian PM_2.5_ involved in either the induction or the inhibition of cell death quantified by different parameters of apoptosis and delineated the mechanism underlying this effect in 16HBE human bronchial epithelial cells. They found that experiments performed with different PM_2.5_ compounds suggest that endotoxins, as well as CB, do not participate to the anti-apoptotic effect of PM_2.5_. Instead, the water-soluble fraction, washed particles, and organic compounds (such as PAH) could mimic this anti-apoptotic activity. Finally, the activation or silencing of the aryl hydrocarbon receptor (AhR) may involve the molecular mechanism of the anti-apoptotic effect of PM_2.5_ at the mitochondrial checkpoint of apoptosis. In conclusion, the PM_2.5_-antiapoptotic effect in addition to the well-documented inflammatory response might explain the maintenance of a prolonged inflammation state induced after pollution exposure, and might delay repair processes of injured tissues [29]. 

Longhin et al. examined the role of PM_2.5_ organic fraction versus washed PM on the cell cycle alterations, and the results have shown that the metabolic activation of PM_2.5_ organic chemicals cause damages to DNA and the spindle apparatus, and G2/M arrest and augmented ROS formation follow it, while washed PM had no such effects [30].

Borgie et al. compared genotoxic/epigenotoxic effects of PM between urban and rural site exposure within a human bronchial epithelial cell line (BEAS-2B). They concluded that inorganic and organic contents of an urban PM (2.5–0.3 μm) sample increased the phosphorylation of H2AX, the telomerase activity, and the miR-21 up-regulation in a dose-dependent manner. Furthermore, urban PM (2.5–0.3 μm) significantly increased the expression of CYP1A1, CYP1B1, and AhRR genes. The variable concentrations of organic compounds and transition metals detected in the PM (2.5–0.3 μm) samples might lead to cumulative DNA damage, which is critical for carcinogenesis [31]. Oh et al. examined the genotoxicity of PM_2.5_ collected from the traffic area in Suwon City, Korea, using cultured BEAS-2B as a model system. Their study suggested that the PM_2.5_ has genotoxic effects and that reactive oxygen species may play a distinct role in these effects. In addition, aliphatic/chlorinated hydrocarbons, PAH/alkyl derivatives, and nitro-PAH/ketones/quinines may be important causative agents of the genotoxic effects [31].

Saint-Georges et al. were interested in genomic alterations after human epithelial lung cells (L132) exposure to air pollution PM in the short-term. In agreement with the current literature, micro satellite (MS) alterations might depend on the ability to induce oxidative stress by dPM, PM, or B[a]P, thereby altering enhancing DNA recombination rates, altering DNA polymerase enzymes, and inhibiting DNA repair enzymes. Hence, they concluded that a crucial underlying mechanism could be the dramatic MS alterations in 3p chromosome multiple critical regions for genomic alterations, which proceeded the lung toxicity in PM-exposed target L132 cells [31].

Lepers et al. analyzed the association between the chemical component of PM samples and bulky DNA adduct formation by examining catalytic activities and CYP1A1 and CYP1B1 genes induction on BEAS-2B. Their data highlight that PM content in PAHs is only partly explained by bulky DNA adduct formation, and suggest that inorganic compounds such as iron support CYP activity to promote bulky DNA adduct formation [32]. Leung et al. examined fifteen PAHs in PM_2.5_ samples in five different cities, and the biological effects of organic extracts were assayed using the BEAS-2B. In the cell culture study, transcript levels of pro-inflammatory cytokine interleukin-6 (IL-6), CYP1A1 and CYP1B1 were found to be induced in the treatment. The cells exposed to extracts from Xian and Beijing demonstrated significant migratory activities, indicating a sign of increased tumorigenicity [32]. Dergham et al. expected to differentiate adverse health effects respective in BEAS-2B of PM_2.5_−0.3 samples produced in rural, urban, or industrial surroundings in vitro. The results show that organic chemicals adsorbed on the three PM_2.5_−0.3 samples (i.e., PAHs) were able to induce the gene expression of xenobiotic-metabolizing enzymes (i.e., NADPH-quinone oxidoreductase-1, and to a lesser extent cytochrome P4501A1 and 1B1). Moreover, intracellular reactive oxygen species within BEAS-2B cells exposed to the three PM (2.5−0.3) samples induced oxidative damage (i.e., 8-hydroxy-2′-deoxyguanosine formation, malondialdehyde production, and/or glutathione status alteration). There were also statistically significant increases of the gene expression and/or protein secretion of inflammatory mediators (i.e., notably IL-6 and IL-8) [33].

Baulig et al. detected candidate inflammatory genes exhibiting transcriptional modifications following urban PM_2.5_ exposure in bronchial epithelial cells. The results show that organic extract seemed to be involved in the expression and secretion of amphiregulin, whereas Growth-regulated protein alpha (GRO-alpha) release was induced by both the aqueous and organic extracts. In conclusion, Paris PM_2.5_ increased protein and mRNA expression of GRO-alpha and AR involved in bronchial remodeling and the chemoattraction process in bronchial epithelial cells, respectively [34]. Lauer et al. utilized BEAS2B to assess the effects of airborne PAHs on biological activities associated with specific biological pathways linked to airway diseases. Results demonstrated that these pathways were involved in oxidative stress (HMOX-1, NQO-1, ALDH3A1, AKR1C1), inflammatory cytokine production (IL-6, IL-8), and aryl hydrocarbon receptor (AhR)-dependent signaling (CYP1A1) [35]. Maciejczyk et al. investigated the sources and individual components of ambient PM_2.5_ that are responsible for the induced cellular response on human bronchial epithelial cells. Nuclear factor kappa B (NF-κB) was selected as a monitor of cellular stress response that followed the exposure to concentrated ambient particles (CAPs). Among the individual components, Ni and V are highly correlated with NF-κB [36]. Yang et al. found that BEAS-2B cells released IL-8, IL-1, and sICAM-1 after exposure to extractable organic matter (EOM) of PM_2.5_. Additionally, the cytokines released could advance lymphocyte T proliferation and promote the expression of CD25, consequently inspiring the airway’s high inflammatory process response [37]. Rodríguez-Cotto et al. collected and examined PM_2.5_ from urban and industrialized regions in Rio de Janeiro in human bronchial epithelial cells, and found that PM_2.5_ aqueous extracts from industrialized regions decreased the release of IL-6 and IL-8. Zinc concentration was higher at the industrial and rural reference sites, although metals were not associated with cytokine changes [34].

According to the literature above, the influence of PM_2.5_ extracts on the airway epithelial cells varies. Organic matter can influence key enzyme expression, thus affecting DNA synthesis and repair. Organic extracts also play an important role in inflammation, oxidative stress, cell differentiation, cell cycle regulation, and signaling pathways. Water-soluble components activate ROS and release inflammatory cytokines, and are also associated with cell apoptosis and genetic toxicity. The transition metals are associated with inflammation and genetic toxicity. Extract mixture components caused different damage based on different constituents.

### 3.4. Toxicity of Co-Culture Cell Model

The major limitation of in vitro studies is that cells have been removed from their normal environment: most in vitro studies are carried out using monoculture systems, thereby excluding neighboring cells with which to interact, yet intercellular signaling is central to tissue and organ homeostasis [38]. This is especially true for freshly isolated cells, where the multidimensional structure and the interplay of the different cell types which normally form a tissue are preserved [38,39,40]. In view of the complex environment of the human organism, the use of only one cell type is too far from reality. Hence, the development of cell co-culture systems in vitro as a strategy for a near-realistic exposure to air pollution PM will be a very useful tool to better conserve the existing cell interactions between the neighboring cell types having direct or indirect contact with deposited PM.

Abbas et al. tried to identify the mechanisms of adverse health effects induced by the VOC and/or PAH components of air pollution PM through in vitro human AM and in an L132 cell co-culture model. They found that in human AM in mono and co-culture, and in L132 cells in monoculture, the gene expression of CYP2E1, CYP1A1, GST-p1, NQO1, and/or GST-l3 increased. However, the use of L132 cells in mono- and co-culture showed quite different outcomes: the pattern of VOC and/or PAH-metabolizing enzymes was induced by PM in L132 cells in monoculture, while remaining almost unaffected in co-culture with AM. Taken together, these results emphasized the key role of human AM in the defenses of PM-exposed human lung from external injuries, notably retaining PM through their higher capacity, and indicated that coated-VOC and/or PAH penetrate into and remain within cells through the physical vector of PM carbonaceous cores, which enabled them to exert a longer toxicity [41]. 

André et al. studied the genotoxic and mutagenic properties of thermally desorbed PM_2.5_ (dPM) collected in Dunkerque (France) using A549 cells and alveolar macrophages (AMs). The formation of 8-hydroxy-2’-deoxyguanosine adducts was observed in A549 cells for PM and dPM in a dose-dependent manner; the effects, probably to some extent, were attributed to the carbonaceous cores. As a result, bulky DNA adducts were found only in AMs after PM and dPM exposure. In conclusion, using relevant exposure models, a suspension of PM_2.5_ was found to induce a combination of DNA interaction mechanisms which could contribute to the induction of lung cancer in exposed populations [42].

Abbas et al. studied the metabolic activation of PAHs coated on PM_2.5_ carbonaceous cores and PAH-DNA bulky stable adduct patterns in human lung epithelial L132 cells and/or human alveolar macrophages in mono- and co-cultures. They found that CYP1A1 gene expression was induced by PAH within air pollution PM_2.5_, but not in L132 cells. However, despite this, PAH-DNA bulky stable adducts were not observed in human AM/L132 cell co-cultures exposed to dPM_2.5_ or PM_2.5_; reliably quantifiable PAH-DNA bulky stable adducts were observed only in L132 cells from human AM/L132 cell co-cultures exposed to B[a]P. Taken together, these results support the exertion of genotoxicity of highly reactive B[a]P-derived metabolites produced not only in primary target human AM, but also in secondary target L132 cells [38].

Gualtieri et al. found that winter PMs had higher levels of PAHs than the summer samples. The PM toxicity was tested in the human pulmonary epithelial cell lines BEAS-2B and A549. The winter PM_2.5_ in the BEAS-2B cells reduced proliferation due to a mitotic delay/arrest, while no such effects were observed in the A549 cells. These results underline that the in vitro responsiveness to PM may be cell line-dependent, and suggest that the different PM properties may trigger different endpoints, such as inflammation, perturbation of the cell cycle, and cell death [43].

Long et al. researched the response of cultures of human T-cells and monocyte-derived macrophages (MDMs) to Synthetic C and C/Fe particulates (1 µm) in order to study the damage of PM_2.5_ carbon particles and iron to the cell. The results showed that T-cells failed to show ultrastructural changes and ingest particles, and the MDMs ingested both types of particles avidly. In contrast, those receiving C particulates showed only ultrastructural changes associated with cell activation; those receiving C/Fe particulates showed evidence of clustering and coalescence of particulates by 24 h. The bioactivity of the C/Fe particulates was demonstrated by treated MDMs by an increase in oxidative burst. Similar cells exposed to C particulates showed no increase in this regard. The synthetic C/Fe particulates also produced hydroxyl radicals on exposure to hydrogen peroxide. We hypothesize that the formation of intracellular ROSs is responsible for the ultrastructural changes observed. Results of these studies demonstrate that particle-induced ultrastructural changes depend on phagocytosis and suggest that among respirable particulates of similar size, biological activity can vary profoundly as a function of particulate physicochemical properties [44]. The related studies on the toxicity of co-culture cell models above have shown that the in vitro cytotoxicity to PM may be cell line-dependent, and the different PM properties with various physical and chemical properties which are key factors of biological effects may trigger different endpoints, such as inflammation, perturbation of the cell cycle, and cell death.

### 3.5. Toxicity Studies on Vascular Endothelial Cells (VEC)

Vascular endothelial cells are a vertically arranged layer of flat cells on the surface of blood vessel linings with active secretion and metabolism functions which are involved in a variety of physiological regulations, such as maintaining blood volume, adjusting blood pressure, resisting thrombosis [45], etc. Their dysfunction is also the pathological basis of many cardiovascular diseases, such as high blood pressure, diabetes, etc. Wei et al. investigated the hypothesis that urban fine PM_2.5_ particles could cause cytotoxicity via oxidative stress in human umbilical vein endothelial cells, EA.hy926. All of the samples were highly enriched in Fe, Zn, and Pb. EA.hy926 cells were exposed to a PM_2.5_ suspension, and water-soluble and water-insoluble fractions, respectively, increasing reactive oxygen species, mitochondrial transmembrane potential disruption, cell death, and NF-κB activation. These results suggest that each fraction of PM_2.5_ has the potency to cause oxidative stress in endothelial cells, generated through the PM_2.5_-mediated mitochondrial apoptotic pathway, which may induce direct interaction between metal elements and endothelial cells [45,46]. Hirano et al. addressed the cytotoxicity and oxidative stress potency of organic extracts of urban fine particles (OE-UFP) and diesel exhaust particles (OE-DEP) in rat heart microvessel endothelial (RHMVE) cells. OE-DEP contains more organic components than OE-UFP, and is comprised of aliphatic and aromatic hydrocarbons, quinones, aldehydes, and heterocyclic organic compounds. The mRNA levels of antioxidant enzymes such as thioredoxin peroxidase 2 (TRPO2), heme oxygenase-1 (HO-1), glutathione S-transferase P subunit (GST-P), and NADPH dehydrogenase (NADPHD) were quantitated by Northern analysis. All of those mRNA levels increased dose-dependently with OE-DEP, and HO-1 mRNA showed the most marked response to OE-DEP. The mRNA levels of antioxidant enzymes and heat shock protein 72 (HSP72) in OE-DEP-exposed cells were higher than those of OE-UFP-exposed cells as compared at the same concentration [46].

Long studied the transcription levels of HO-1 and HSP72 in OE-DEP- and OE-UFP-exposed cells. Those results suggest that the organic fraction of particulate materials in the urban air has the potency to cause oxidative stress to endothelial cells, and may be implicated in cardiovascular diseases through functional changes of endothelial cells [43]. Furuyama et al. also examined the production of heme oxygenase-1 and factors related to the fibrinolytic function by rat heart microvessel endothelial cells exposed to organic extracts of diesel exhaust particles and urban fine particles to investigate the direct effects of these soluble organic fractions in these PM on the fibrinolytic function of endothelial cells. The results suggest that exposure to the soluble organic fraction of PM and DEP induced oxidative stress and reduced the PAI-1 production of endothelial cells [43]. In conclusion, metal elements may induce direct interaction between oxidative stress in endothelial cells. The soluble organic fraction of PM and DEP induced oxidative stress and the mRNA levels of antioxidant enzymes. Those results suggest that the organic fraction of particulate materials in the urban air has the potency to cause oxidative stress to endothelial cells. functional changes of endothelial cells and may be implicated in cardiovascular diseases.

### 3.6. Toxicity Studies on Other Cells

In addition to the above cell lines, scholars also studied the cytotoxicity of PM_2.5_ in human hepatoma cells, HepG2; human diploid lung fibroblasts, HEL; BALB/c 3T3; MCF-7; human fibroblasts; human embryonic lung fibroblasts (HEL12469), and so on. Results show that PM_2.5_ caused different extents of damage to these cell lines. Hanzalova et al. investigated the role of oxidative damage in the mechanism of action of selected individual carcinogenic PAHs (c-PAHs: benzo[*a*]pyrene, B[a]P, dibenzo[*a*,*l*]pyrene, DB[*a*,*l*]P), an artificial mixture of c-PAHs (c-PAHs mix), and EOM from urban air PM by two cell lines. Their results demonstrate the ability of EOM to induce oxidative damage to DNA and lipids after 24 h of treatment, and to proteins after 48 h, while the effect of c-PAHs was substantially less in HepG2 cells. In contrast, the induction of oxidative stress by c-PAHs and EOM in HEL cells was weak [47].

Vaccari et al. tested PM_2.5_ samples’ organic extracts in the BALB/c 3T3 CTA, and a dose-related toxic effect was observed in the exposed cells. The toxicity related with the sampling season and sampling site was confirmed by modulated gene pathways. The functional analysis of the KEGG (Kyoto Encyclopedia of Genes and Genomes)’s pathways showed modulation of several gene networks which are related to inflammation and oxidative stress. The samples did not induce cell transformation in the treated cells, but gene pathways related to the onset of cancer were modulated as a consequence of the exposure [47]. Líbalová et al. collected PM_2.5_ in four localities of the Czech Republic (Ostrava-Bartovice, Ostrava-Poruba, Karvina, and Trebon) which differed in the extent and sources of air pollution. HEL12469 were treated with the same four EOMs to assess changes in the genome-wide expression profiles compared to DMSO-treated controls. The results have shown the metabolism of xenobiotics by cytochrome P450 exhibited the strongest up-regulation in all four localities, and CYP1B1 had a major contribution to the up-regulation of this pathway. Other important deregulated pathways in all four localities were ABC transporters (involved in the translocation of exogenous and endogenous metabolites across membranes and DNA repair), the Wnt and TGF-β signaling pathways (associated particularly with tumor promotion and progression), steroid hormone biosynthesis (pathways involved in the endocrine-disrupting chemicals activity), and glycerolipid metabolism (pathways associated with the lipids with a glycerol backbone), which suggested a prominent role of the activation of aryl hydrocarbon receptor-dependent gene expression [48].

Musafia-Jeknic et al. used SRM 1649a representative examples of carcinogenic PAHs, a number of natural matrix standard reference materials, B[a]P and DB[*a*,*l*]P, to understand the metabolic activation and DNA binding of these PAHs within complex mixtures [49]. Cell line MCF-7 was treated with SRM 1649a alone, or SRM 1649a with either B[a]P or DB[*a*,*l*]P for 24–120 h. Cytochrome P450 (CYP) enzyme activity, CYP1A1 and CYP1B1 protein expression, and PAH-DNA adduct were determined. Induction of CYP1A1 and CYP1B1 protein expression was observed in cells treated with BP alone or in co-treatments of SRM 1649a and B[a]P or DB[*a*,*l*]P. These data demonstrate that the components of SRM 1649a inhibit the activation of B[a]P or DB[*a*,*l*]P by CYP enzymes in DNA adduct formation. It also suggests that the carcinogenic activity of PAH within a complex mixture may vary with the composition and activation of the components present in the complex mixture [50,51]. Mahadevan et al. conducted similar research and observed that RM 1649a decreased the total level of BP-DNA adducts in comparison with B[a]P alone. No significant difference in adduct levels was observed in response to either DB[*a*,*l*]P alone or in combination with SRM 1649a. These results provide a transcriptional signature for chemical carcinogen exposure. In addition, they suggest that a major factor in the carcinogenic activity of PAH within complex mixtures is their ability to promote or inhibit the activation of carcinogenic PAH by the induction of CYP enzymes [51].

Karlsson et al. investigated the genotoxicity of PM in relation to particle–cell interactions to study the effect of the removal of DNA-damaging substances by the extraction of PM with different solvents from human fibroblasts. The genotoxicity of PM was caused both by adduct-forming poly aluminium chloride (PACs) and oxidizing substances, as well as the insoluble particle core. This study showed that all these factors together contribute to explaining the mechanisms of PM genotoxicity [52]. Jan Topinka et al. observed distinct genotoxic and cytotoxic potencies of PAHs. Contrary to that, chrysene, benz[*a*]anthracene, dibenzo[*a*,*h*]anthracene, and benzo[*b*]fluoranthene induced only low amounts of DNA adduct formation and minimal apoptosis, with no significant effects on p53 phosphorylation. The present data show that in model cell lines sharing phenotypic properties with oval cells, PAHs can be efficiently metabolized to form ultimate genotoxic metabolites. Liver progenitor cells could thus be susceptible to this type of genotoxic insult, which makes the WB-F344 cell line a useful tool for studies of the genotoxic effects of organic contaminants in liver cells. Their results also suggest that—unlike in mature hepatocytes—CYP1B1 might be a primary enzyme responsible for the formation of DNA adducts in liver progenitor cells [53]. Tarantini et al. cultured human hepatocytes and treated them with either pure B[a]P or particulate matter extracted from air samples collected in an urban industrial site or in a metallurgic plant. Comparison with the effect of the reconstituted PAH fraction of the mixtures concluded that the induction of strand breaks results from the action of other components of the samples. In addition, a 30% potentialization and a 90% inhibition in the level of DNA adducts with respect to exposure to pure B[a]P were observed for cells exposed to industrial and urban mixtures, respectively. These results contrast with the six-fold enhancement in the yield of broparoestrol (BPDE) adducts in cells exposed to the reconstituted PAH fraction with respect to pure BaP [47].

Andrysík et al. used an organic extract of the urban dust standard reference material SRM1649a as a model mixture to study a range of toxic effects related to DNA damage and AhR activation in the liver epithelial WB-F344 cells model. They found that this extract and its neutral and polar fractions were potent inducers of a range of AhR-mediated responses, including induction of the AhR-mediated transcription—such as cytochrome P450 1A1/1B1 expression—and AhR-dependent cell proliferation. Importantly, these toxic events occurred at doses one order of magnitude lower than the DNA damage. The AhR-mediated activity of the neutral fraction was linked to PAHs and their derivatives, as polychlorinated dibenzo-p-dioxins, dibenzofurans, and biphenyls were only minor contributors to the overall AhR-mediated activity. Taken together, more attention should be paid to the AhR-dependent non-genotoxic events elicited by urban PM constituents, especially PAHs and their derivatives [54]. On the basis of the impact of PM_2.5_ on the liver, related studies have shown that PAH with other ingredients of PM can cause genetic toxicity in liver cells, and PM_2.5_ organic mixture composition promotes and inhibits the carcinogenic potential of PAHs through the induction of the CYP enzyme. This conclusion was consistent with the cell toxicity of the PM_2.5_ organic composition for cell line MCF-7. The genetic toxicity depends on the effect of the mixture composition and activation status. Several studies of PM components’ toxicity in human fibroblasts have shown that all components of PM can cause genetic toxicity, adjust cell signaling pathways, and affect protein and lipid expression.

Above all, there are no PM_2.5_ components for which there is unequivocal evidence of zero health impact, including oxidative stress, inflammation, genetic toxicity, and activation of signaling pathways. PM_2.5_ had similar cytotoxicity (e.g., cell viability reduction, oxidative damage, inflammatory effects and genetic toxicity) on different types of cells. However, each group of components have not clearly shown consistent associations with specific health effects (Table 1). Toxicity of a mixed composition depends on its content, which differs greatly from the single components in mixed ingredients. In addition to this, single or similar composition models for single-cell toxicity studies are not enough to explain the mechanism of health damage. By comparing different cell toxicity reactions, we may be safe to conclude that the target cell toxic effects caused by fine particulate matter are specific, to a certain extent, and the results can partly explain that PM_2.5_ has a harmful effect to the body’s organs.

## 4. Discussion

The toxicology of PM_2.5_ on cells and its involved mechanisms is still not clear enough. Therefore, there are several issues to be resolved. The PM_2.5_ components vary considerably depending on season, region, and sources, which made the results of studies have low comparability; thus, it is difficult to shape systematic study conclusions, so we should strengthen the research on the characteristics of time and space distribution of PM_2.5_ and their physicochemical characteristics. Additionally, it is also important to analyze the toxic effects of PM_2.5_ with different physical and chemical compositions on cells at different stages, locations, and levels. Second, whether the toxicity of PM_2.5_ is the combined effect of each component or a single component is not clear. Therefore, except for researching PM_2.5_ in a single composition for cell toxicity, we should also consider the interaction effects between different PM_2.5_ components, as well as the combined effect with other atmospheric pollutants. Third, the method of cytotoxicity study in in vitro cells should be developed and refined. For a near-realistic exposure to PM_2.5_, co-culture systems can be used to better conserve the existing cell interactions between neighboring cell types. In order to further clarify the toxicity of PM_2.5_ in different target cell lines, we can compare a variety of cytotoxic effects. Fourth, current studies tend to use high doses and acute toxicity tests taking the place of low dose and long-range research to perform the toxicity of PM_2.5_ in in vitro cell cultures, but the real exposure concentration is generally lower than the concentration of animal experiments by a significant margin. Low-dose, low concentration, and long exposure studies should be further conducted, along with chronic toxicity studies in order to better elaborate the impact of PM_2.5_ exposure on populations or individual health and its related mechanisms. Finally, based on the results of cell and animal experiments on the biological effects of PM_2.5_ from the aspects of genetic, immune, and other carcinogenic effects, the interaction mechanisms of a variety of acute and chronic disease occurrences and developments were evaluated, and provide a scientific basis for proposing PM_2.5_ biological evaluation methods of pollution and the comprehensive evaluation of air quality.

## 5. Conclusions

Data in the present study reveal that the toxicity of a mixed composition differs greatly from the single ingredients in mixed component and the target cells. The cytotoxic responses caused by PM_2.5_’s each grouped components have not clearly shown a consistent association with specific health effects. In addition to this, interaction between components was clearly observed. Our results may be beneficial for providing new targets for drugs for the treatment of PM_2.5_-related diseases.

## Figures and Tables

**Table 1:** Main cytotoxicity of different PM_2.5_ components. PAH: polycyclic aromatic hydrocarbons.

		Main Cytotoxicity
Inorganic components	Metals	Oxidative damage
	Nonmetals	cell viability reduction
	Transition metals	Oxidative damage inflammatory effects
Organic components	Carbons	inflammatory effects
	PAHs	Genetic toxicity
	Quinones	Genetic toxicity inflammatory effects
Aqueous components	-	cell viability reduction DNA damage Apoptosis

## Abbreviations

HPLS-FD: High performance liquid chromatography fluorescence detection
GC-MS: gas chromatography-mass spectrometry
ICP-MS: inductively coupled plasma mass spectrometer
